# Pharmacovigilance study of BCR-ABL1 tyrosine kinase inhibitors: a safety analysis of the FDA adverse event reporting system

**DOI:** 10.1186/s40360-024-00741-x

**Published:** 2024-02-23

**Authors:** Dehua Zhao, Xiaoqing Long, Jisheng Wang

**Affiliations:** Department of Clinical Pharmacy, The Third Hospital of Mianyang (Sichuan Mental Health Center), 621000 Mianyang, Sichuan People’s Republic of China

**Keywords:** BCR-ABL1 TKIs, Adverse events, Pharmacovigilance, FAERS, Disproportionality analysis

## Abstract

**Background:**

With the increased use of BCR-ABL1 tyrosine kinase inhibitors (TKIs) in cancer patients, adverse events (AEs) have garnered considerable interest. We conducted this pharmacovigilance study to evaluate the AEs of BCR-ABL1 TKIs in cancer patients using the Food and Drug Administration Adverse Event Reporting System (FAERS) database.

**Methods:**

To query AE reports from the FAERS database, we used OpenVigil 2.1. Descriptive analysis was then employed to describe the characteristics of TKIs-associated AE reports. We also utilized the disproportionality analysis to detect safety signals by calculating the proportional reporting ratio (PRR) and reporting odds ratios (ROR).

**Results:**

From the FAERS database, a total of 85,989 AE reports were retrieved, with 3,080 significant AE signals identified. Specifically, imatinib, nilotinib, dasatinib, bosutinib, and ponatinib had significant AE signals of 1,058, 813, 232, 186, and 791, respectively. These significant signals were further categorized into 26 system organ classes (SOCs). The AE signals of imatinib and ponatinib were primarily associated with general disorders and administration site conditions. On the other hand, nilotinib, dasatinib, and bosutinib were mainly linked to investigations, respiratory, thoracic and mediastinal disorders, and gastrointestinal disorders, respectively. Notably, new signals of 245, 278, 47, 55, and 253 were observed in imatinib, nilotinib, dasatinib, bosutinib, and ponatinib, respectively.

**Conclusions:**

The results of this study demonstrated that AE signals differ among the five BCR-ABL1 TKIs. Furthermore, each BCR-ABL1 TKI displayed several new signals. These findings provide valuable information for clinicians aiming to reduce the risk of AEs during BCR-ABL1 TKI treatment.

**Supplementary Information:**

The online version contains supplementary material available at 10.1186/s40360-024-00741-x.

## Introduction

Chronic myeloid leukaemia (CML) is a myeloproliferative neoplasm caused by the presence of the Philadelphia chromosome [1]. The Philadelphia chromosome contains a BCR-ABL1 fusion gene that encodes a constitutively active cytoplasmic tyrosine kinase. This kinase activates various signals involved in promoting the proliferation and survival of myeloid progenitor cells [2]. Therefore, the BCR-ABL1 kinase is the key target for CML therapy. Several BCR-ABL1 tyrosine kinase inhibitors (TKIs) have been developed and approved for the treatment of CML [3]. In addition to being used to treat CML, these TKIs are also used to treat other malignancies, such as acute lymphoblastic leukemia (ALL), dermatofibrosarcoma protuberans (DFSP), and gastrointestinal stromal tumors (GIST) [2–4]. The first-generation TKI is imatinib, while dasatinib, nilotinib, and bosutinib are second-generation TKIs, and ponatinib is the third-generation TKI [3, 4]. These TKIs inhibit the activity of BCR-ABL tyrosine kinase by binding to it in an inactive form, leading to the death of tumor cells. Although BCR-ABL1 TKIs have significantly improved the survival of patients with CML, they are not without adverse events (AEs) [5, 6]. AEs induced by BCR-ABL1 TKIs may reduce therapeutic adherence; therefore, pharmacovigilance studies of these drugs are essential for successful CML treatment.

Most of the efficacy and safety data of BCR-ABL1 TKIs are obtained from clinical trials. However, clinical trials have limitations in fully reflecting safety data from real clinical settings due to strict inclusion criteria, relatively small sample size, or limited follow-up durations. Therefore, there may be unknown adverse reactions occurring in real-world clinical settings. The Food and Drug Adverse Event Reporting System (FAERS) is one of the largest spontaneous reporting database in the world, providing sufficient data to verify and supplement the findings of clinical trials [7]. As the FAERS database is publicly available and reflects complete AE reports in real-world clinical settings, it is widely used to detect potential drug-associated AEs [8]. In this study, we conducted a pharmacovigilance analysis using the FAERS database to evaluate and compare the safety of BCR-ABL1 TKIs.

## Materials and methods

### Data sources and data collection

The data collection for this study utilized OpenVigil 2.11, which allowed us to retrieve the FAERS data from drug approval up to the third quarter of 2022 (Table 1). We collected specific clinical characteristics for each adverse event (AE) report, including individual safety reports (ISR), outcome, drug name, role code, dosage, indication, event, case ID, gender, reporter country, and age in the report. Given that the FAERS database is a compilation of submissions from various sources, duplicates can be found within the dataset. To address this, we utilized the case ID and ISR as key filters, choosing the higher ISR in cases where the case ID matched. Furthermore, in order to minimize confounding effects, preferred terms (PTs) associated with indication, off-label use, and product use issues were excluded from the analysis.

**Table 1:** Information of BCR-ABL1 TKIs

Generic name	Brand name	Approval date	Indications
Imatinib	Gleevec	2001.05.10	CML, ALL, MDS, MPD, ASM, HES, CEL, DFSP, GIST
Dasatinib	Sprycel	2006.06.28	CML, ALL
Nilotinib	Tasigna	2007.10.29	CML
Bosutinib	Bosulif	2012.09.04	CML
Ponatinib	Iclusig	2012.12.14	CML, ALL

ALL, acute lymphoblastic leukemia; MDS, myelodysplastic diseases; MPD, myeloproliferative diseases; ASM, aggressive systemic mastocytosis; HES, hypereosinophilic syndrome; CEL, chronic eosinophilic leukemia; DFSP, dermatofibrosarcoma protuberans; GIST, gastrointestinal stromal tumors

### Adverse events and drug identification

We employed both the generic name and brand name, including “imatinib”, “gleevec”, “nilotinib”, “tasigna”, “bosutinib”, “bosulif” “dasatinib”, “sprycel”, “ponatinib”, and “iclusig”, to identify AE records associated with the target drugs. Our search was specifically focused on AE reports in which the drug was considered the primary suspect, aiming to improve accuracy. The AEs were coded using the PTs according to the Medical Dictionary for Regulatory Activities (MedDRA) terminology. Additionally, we utilized MedDRA (version 22.1) to classify the AEs in each report into the corresponding system organ class (SOC) levels.

### Statistical analysis

A descriptive analysis was conducted to summarize the clinical characteristics of the AE reports, including the event, outcome, gender, age, and reporting country. To study the correlation between the target drug and the target AEs, a disproportionality analysis was employed. The reporting odds ratio (ROR) and proportional reporting ratio (PRR) were calculated to generate signals of disproportionate reporting. The specific algorithm for the disproportionality analysis was outlined in Table 2 [9], while the equations and criteria were listed in Table 3 [9]. A signal is considered significant when both algorithms yield positive results. Furthermore, a higher ROR or PRR value indicates a stronger signal between the drug and AE. It is also important to note that we performed an new signal analysis to identify any new signals associated with the five BCR-ABL1 TKIs. New signals were defined as significant AEs that were not listed in the drug label [10–12]. Additionally, according to the FDA classification, the outcomes of AE reports were grouped into serious or non-serious in FAERS. The serious outcomes included death, life-threatening, disability, initial or prolonged hospitalization, or other serious medical events. In this study, we also presented the AEs with the highest proportion of serious outcomes for each BCR-ABL1 TKI. All data processing and statistical analysis were performed using Microsoft Excel 2019 and SPSS 23.0 statistical software.

**Table 2:** Disproportionality analysis algorithm

Item	Target AEs	Other AEs	Sums
Target drug	a	b	a + b
Other drugs	c	d	c + d
Sums	a + c	b + d	a + b + c + d

**Table 3:** The equations and criteria for the algorithm

Algorithms	Equation	Criteria
ROR	ROR= (a×d) / (b×c)	lower limit of the 95% CI > 1 and a ≥ 3
	95%CI = e^ln(ROR)±1.96 × (1/a+1/b+1/c+1/d)∧0.5^	
PRR	PRR= [a×(c + d)] / [c×(a + b)]	PRR ≥ 2, χ^2^ ≥ 4, and a ≥ 3
	χ^2^= [(a×d − b×c)^2^]×(a + b + c + d) /[(a + b)×(c + d)×(a + c)×(b + d)]	

ROR, reporting odds ratio; PRR, proportional reporting ratio; CI, confidence interval; χ^2^, chi-squared

## Results

### Descriptive analysis

The number of AE reports for imatinib, nilotinib, dasatinib, bosutinib, and ponatinib were as follows: 39,746, 17,351, 19,633, 3,978, and 5,281, respectively. In terms of gender, there were more male patients than female patients. The median age was 60 years with an interquartile range (IQR) of 46 to 70. The majority of AE reports were from North America (51.41%), followed by Asia (15.39%), Europe (10.57%), South America (2.09%), Africa (1.33%), and Oceania (1.19%). The most frequently reported outcomes were other outcomes (27.21%), followed by death (27.03%), hospitalization (15.65%), life-threatening events (1.61%), and disability (0.61%). The characteristics of AE reports for different BCR-ABL1 TKIs were illustrated in Table 4.

**Table 4:** Characteristics of AE reports for different BCR-ABL1 TKIs

Characteristics	Imatinib	Nilotinib	Dasatinib	Bosutinib	Ponatinib	All BCR-ABL1 TKIs
**Number of events**	39,746	17,351	19,633	3978	5281	85,989
**Age (year), n (%)**						
< 18	659 (1.66)	75 (0.43)	270 (1.38)	5 (0.13)	39 (0.74)	1048 (1.22)
18–44	3293 (8.29)	1635 (9.42)	2059 (10.49)	391 (9.83)	565 (10.70)	7943 (9.24)
45–64	6504 (16.36)	3064 (17.66)	4537 (23.11)	1169 (29.39)	916 (17.35)	16,190 (18.83)
65–74	3558 (8.95)	1614 (9.30)	2196 (11.19)	809 (20.34)	498 (9.43)	8675 (10.09)
≥ 75	2961 (7.45)	1129 (6.51)	1509 (7.69)	714 (17.95)	354 (6.70)	6667 (7.75)
Unknown	22,771 (57.29)	9834 (56.68)	9062 (46.16)	890 (22.37)	2909 (55.08)	45,466 (52.87)
Median (IQR)	60 (46, 71)	59 (46, 70)	59 (47, 69)	64 (53, 74)	44 (58, 69)	60 (46, 70)
**Gender, n (%)**						
Female	16,898 (42.51)	7326 (42.22)	9226 (46.99)	1803 (45.32)	2037 (38.57)	37,290 (43.37)
Male	19,419 (48.86)	8310 (47.89)	8745 (44.54)	1770 (44.49)	2523 (47.78)	40,767 (47.41)
Unknown	3429 (8.63)	1715 (9.88)	1662 (8.47)	405 (10.18)	721 (13.65)	7932 (9.22)
**Reported region, n (%)**						
North America	13,109 (32.98)	7120 (41.04)	16,995 (86.56)	3311 (83.23)	3673 (69.55)	44,208 (51.41)
Asia	8279 (20.83)	2992 (17.24)	961 (4.89)	296 (7.44)	702 (13.29)	13,230 (15.39)
Europe	4476 (11.26)	2395 (13.80)	1230 (6.26)	315 (7.92)	677 (12.82)	9093 (10.57)
South America	852 (2.14)	538 (3.10)	220 (1.12)	41 (1.03)	147 (2.78)	1798 (2.09)
Africa	878 (2.21)	153 (0.88)	67 (0.34)	14 (0.35)	30 (0.57)	1142 (1.33)
Oceania	630 (1.59)	241 (1.39)	112 (0.57)	1 (0.03)	42 (0.80)	1026 (1.19)
Unknown	11,522 (28.99)	3912 (22.55)	48 (0.24)	0 (0)	10 (0.19)	15,492 (18.02)
**Outcome of AEs, n (%)**						
Other outcomes	11,433 (28.77)	5249 (30.25)	4586 (23.36)	805 (20.24)	1326 (25.11)	23,399 (27.21)
Death	15,660 (39.40)	3634 (20.94)	1884 (9.60)	375 (9.43)	1694 (32.08)	23,247 (27.03)
Hospitalization (initial or prolonged)	4796 (12.07)	2832 (16.32)	3546 (18.06)	626 (15.74)	1655 (31.34)	13,455 (15.65)
Life-threatening	569 (1.43)	370 (2.13)	339 (1.73)	34 (0.85)	75 (1.42)	1387 (1.61)
Disability	287 (0.72)	145 (0.84)	64 (0.33)	9 (0.23)	18 (0.34)	523 (0.61)
Unknown	7001 (17.61)	5121 (29.51)	9214 (46.93)	2129 (53.52)	513 (9.71)	23,978 (27.88)

### Disproportionality analysis

#### PTs analysis

Two algorithms and their corresponding criteria were used to detect all AE signals of BCR-ABL1 TKIs. The numbers of significant AE signals for imatinib, nilotinib, dasatinib, bosutinib, and ponatinib were as follows: 1,058, 813, 232, 186, and 791. The top 20 most frequently reported AE signals that met the criteria were listed in supplemental Tables 1–5. Among these signals, platelet count decreased was detected in five drugs, while pleural effusion and rash were detected in four drugs. Abdominal pain, death, fatigue, fluid retention, malignant neoplasm progression, myalgia, and thrombocytopenia were detected in three drugs. Abdominal pain upper, anaemia, chest pain, constipation, diarrhoea, drug intolerance, decreased haemoglobin, headache, hospitalisation, neoplasm progression, oedema, pain in extremity, pancytopenia, pyrexia, second primary malignancy, and decreased white blood cell count were detected in two drugs.

**Fig. 1:**
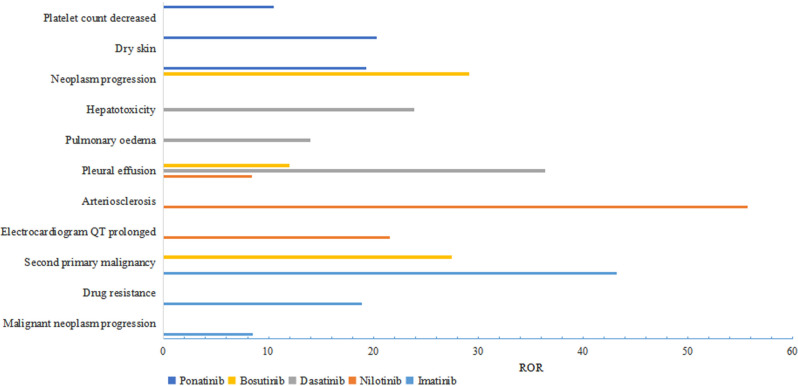
The top 3 strongest signals for different BCR-ABL1 TKIs

The signal values differed among the five BCR-ABL1 TKIs. The top 3 strongest signals for different BCR‐ABL1 TKIs were illustrated in Fig. 1. For imatinib, stronger signals were observed in second primary malignancy (PRR = 42.622, ROR = 43.229), drug resistance (PRR = 18.524, ROR = 18.875), and malignant neoplasm progression (PRR = 8.263, ROR = 8.512). Nilotinib showed stronger signals in arteriosclerosis (PRR = 54.604, ROR = 55.738), prolonged electrocardiogram qt (PRR = 20.873, ROR = 21.613), and pleural effusion (PRR = 8.255, ROR = 8.407). Dasatinib exhibited stronger signals in pleural effusion (PRR = 33.536, ROR = 36.388), hepatotoxicity (PRR = 23.35, ORR = 23.892), and pulmonary oedema (PRR = 13.71, ROR = 14.033). Bosutinib displayed stronger signals in neoplasm progression (PRR = 27.616, ROR = 29.144), second primary malignancy (PRR = 27.086, ROR = 27.459), and pleural effusion (PRR = 11.737, ROR = 12.018). As for ponatinib, stronger signals were observed in dry skin (PRR = 19.227, ROR = 20.299), neoplasm progression (PRR = 18.667, ROR = 19.342), and decreased platelet count (PRR = 10.096, ROR = 10.515).

In terms of serious outcomes, the AE with the highest proportion of serious outcomes was sepsis (92.68%) for imatinib, haemoglobin decreased (65.90%) for nilotinib, pericardial effusion (52.21%) for dasatinib, dehydration (59.65%) for bosutinib, and pneumonia (91.28%) for ponatinib.

#### SOCs analysis

**Fig. 2:**
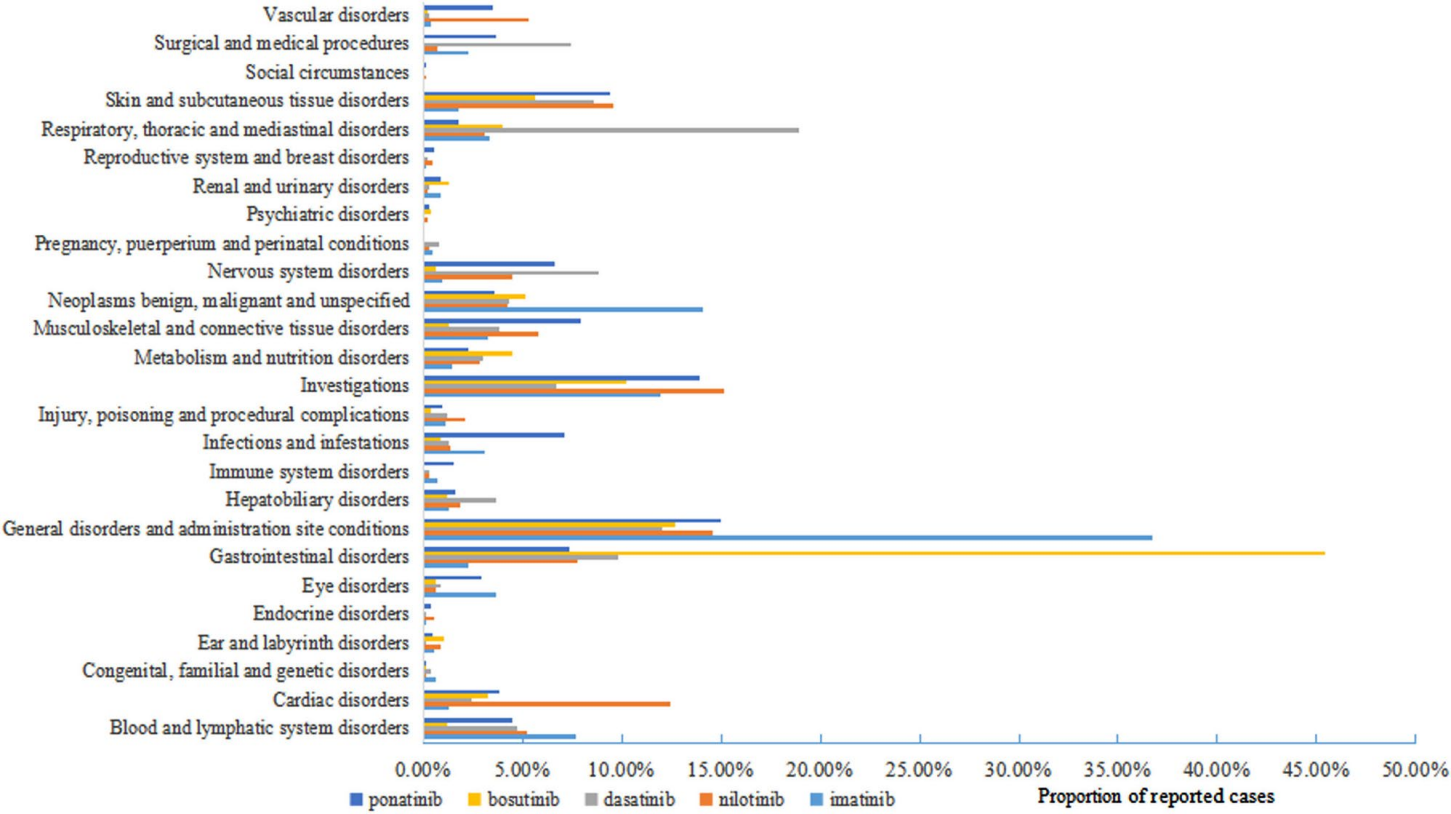
Proportion of reported cases for different BCR-ABL1 TKIs in SOC level

**Fig. 3:**
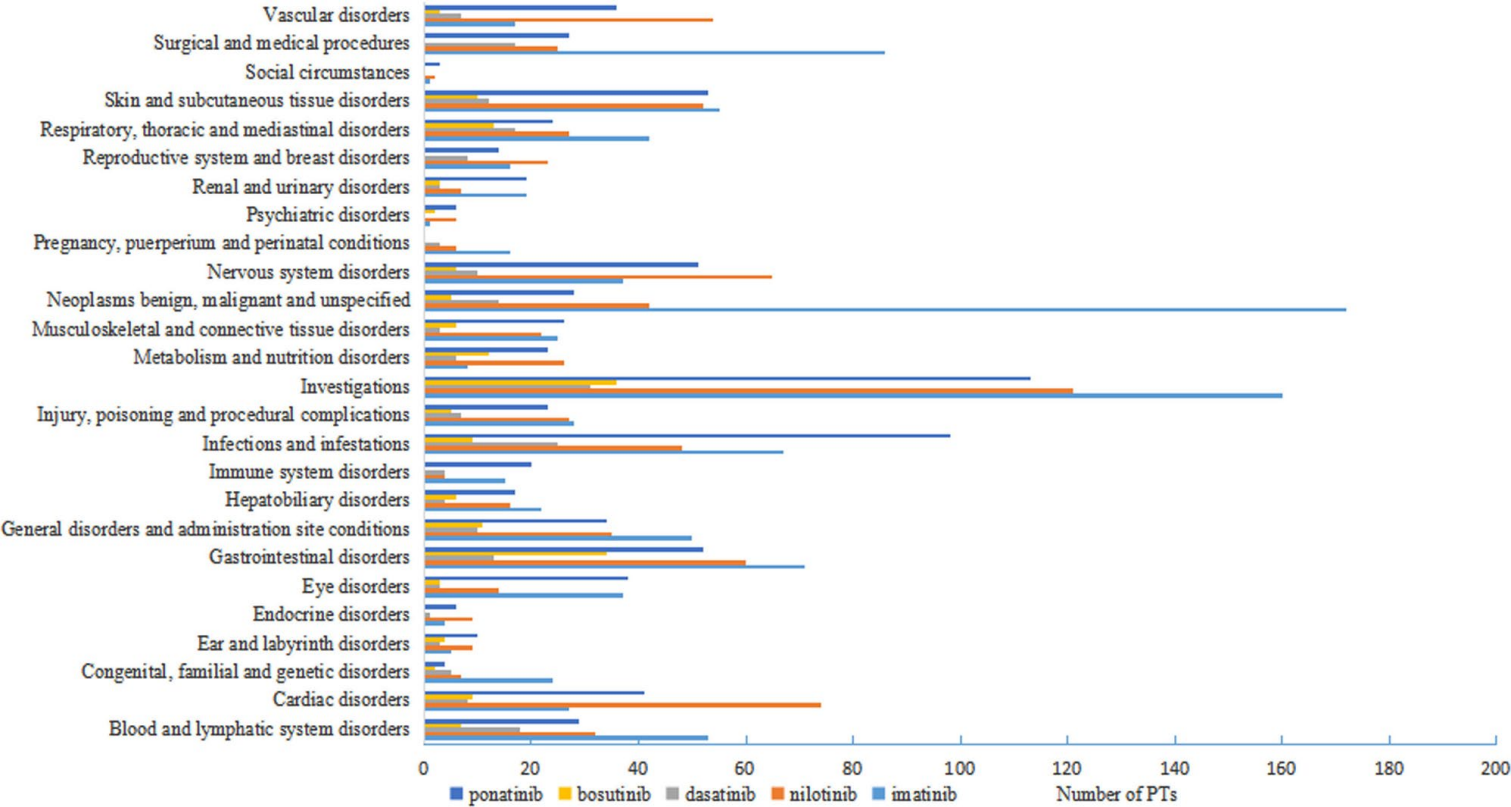
Number of PTs for different BCR-ABL1 TKIs in SOC level

Within the SOC level, 26 SOCs were identified for all AE signals. Imatinib and nilotinib AEs involved 26 SOCs, while dasatinib AEs involved 24 SOCs, bosutinib AEs involved 20 SOCs, and ponatinib AEs involved 25 SOCs. The proportion of reported cases for different BCR-ABL1 TKIs in SOC level were shown in Fig. 2. The number of PTs for different BCR‐ABL1 TKIs in SOCs level were shown in Fig. 3. Moving on to the most commonly reported SOCs for each BCR-ABL1 TKI, imatinib had general disorders and administration site conditions (18,956 cases, 36.73%), neoplasms benign, malignant and unspecified (incl cysts and polyps) (7,252 cases, 14.05%), and investigations (6,148 cases, 11.91%) as the top three SOCs. For nilotinib, the top three SOCs were investigations (5,472 cases, 15.13%), general disorders and administration site conditions (5,279 cases, 14.59%), and cardiac disorders (4,487 cases, 12.40%). Dasatinib had respiratory, thoracic and mediastinal disorders (2,929 cases, 18.95%), general disorders and administration site conditions (1,861 cases, 12.04%), and gastrointestinal disorders (1,518 cases, 9.82%) as its top three SOCs. Bosutinib had gastrointestinal disorders (3,108 cases, 45.39%), general disorders and administration site conditions (870 cases, 12.70%), and investigations (701 cases, 10.24%) as its top three SOCs. Lastly, for ponatinib, the top three SOCs were general disorders and administration site conditions (3,348 cases, 14.97%), investigations (3,105 cases, 13.88%), and skin and subcutaneous tissue disorders (2,097 cases, 9.38%).

Regarding the signal values for each BCR-ABL1 TKI, the strongest signals for imatinib were endocrine disorders (PRR = 14.868, ROR = 14.877), psychiatric disorders (PRR = 10.686, ROR = 10.687), and congenital, familial and genetic disorders (PRR = 10.286, ROR = 10.333). Nilotinib had vascular disorders (PRR = 15.090, ROR = 16.485), social circumstances (PRR = 11.480, ROR = 11.486), and pregnancy, puerperium and perinatal conditions (PRR = 8.737, ROR = 8.777) as its top three strongest signals. The top three strongest signals for dasatinib were hepatobiliary disorders (PRR = 17.245, ROR = 17.661), respiratory, thoracic and mediastinal disorders (PRR = 15.981, ROR = 18.198), and congenital, familial and genetic disorders (PRR = 10.321, ROR = 10.345). For bosutinib, the top three strongest signals were neoplasms benign, malignant and unspecified (incl cysts and polyps) (PRR = 26.822, ROR = 28.802), congenital, familial and genetic disorders (PRR = 12.205, ROR = 12.226), and vascular disorders (PRR = 11.621, ROR = 11.652). Lastly, ponatinib had congenital, familial and genetic disorders (PRR = 22.414, ROR = 22.480), neoplasms benign, malignant and unspecified (incl cysts and polyps) (PRR = 21.780, ROR = 24.245), and social circumstances (PRR = 17.801, ROR = 17.830) as its top three strongest signals.

#### New signals

**Fig. 4:**
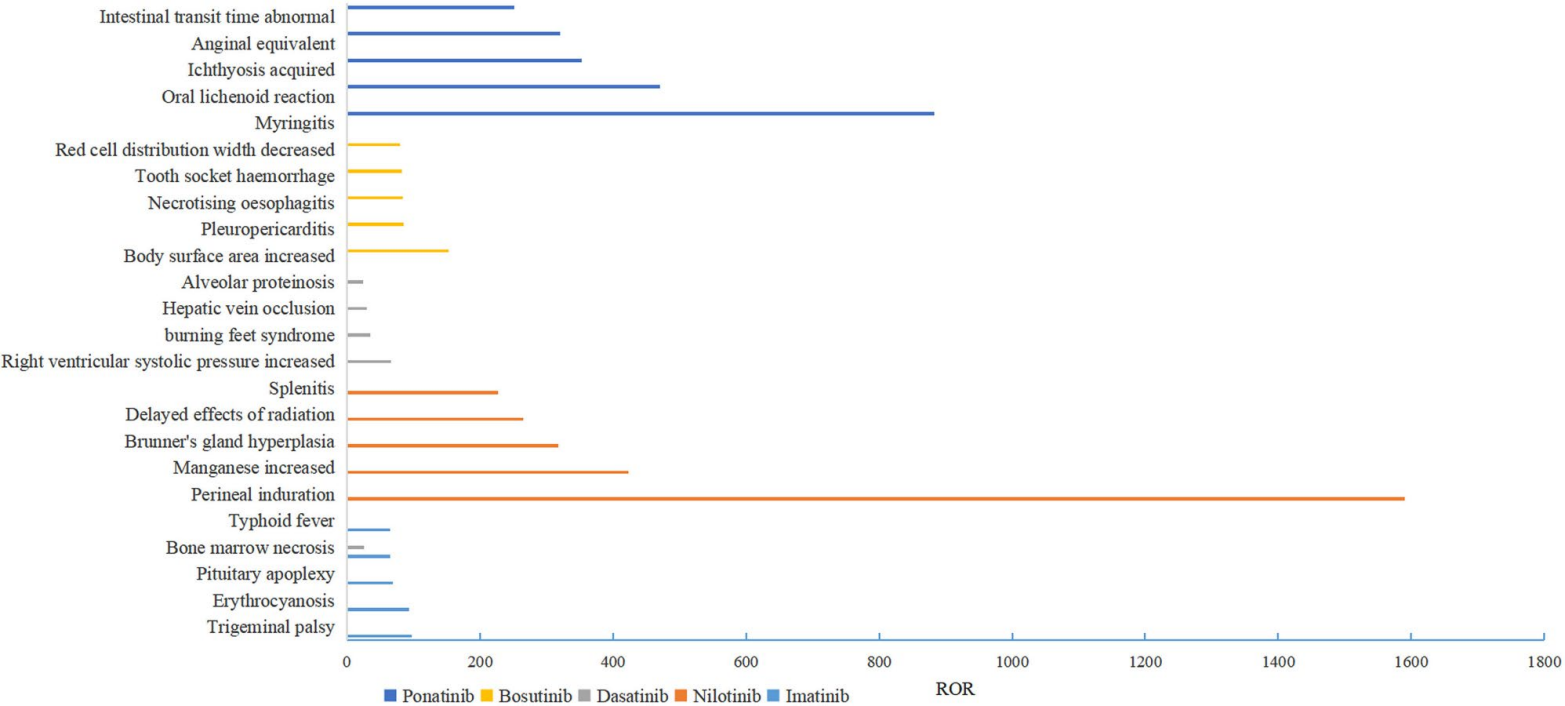
The top 5 strongest signaling new signals for different BCR-ABL1 TKI

New signals were observed for imatinib, nilotinib, dasatinib, bosutinib, and ponatinib, after searching in drug labels. In total, 245 new signals were found for imatinib, 278 for nilotinib, 47 for dasatinib, 55 for bosutinib, and 253 for ponatinib. The new signals for each BCR-ABL1 TKI were listed in supplemental Table 6. The top 5 strongest signaling new signals for different BCR-ABL1 TKI were illustrated in Fig. 4. For imatinib, the top 5 strongest signals were trigeminal palsy (PRR = 70.800, ROR = 96.988), erythrocyanosis (PRR = 69.227, ROR = 94.045), pituitary apoplexy (PRR = 54.653, ROR = 68.978), bone marrow necrosis (PRR = 52.237, ROR = 65.189), and typhoid fever (PRR = 52.210, ROR = 65.152). The top 5 strongest signals for nilotinib were perineal induration (PRR = 398.127, ROR = 1589.733), manganese increased (PRR = 235.927, ROR = 423.949), brunner’s gland hyperplasia (PRR = 199.063, ROR = 317.991), delayed effects of radiation (PRR = 176.945, ROR = 264.968), and splenitis (PRR = 159.251, ROR = 227.458). Dasatinib had the top 5 strongest signals of right ventricular systolic pressure increased (PRR = 58.444, ROR = 65.949), burning feet syndrome (PRR = 32.775, ROR = 34.978), hepatic vein occlusion (PRR = 27.836, ROR = 29.397), bone marrow necrosis (PRR = 24.525, ROR = 25.726), and alveolar proteinosis (PRR = 23.092, ROR = 24.156). The top 5 strongest signals for bosutinib were body surface area increased (PRR = 140.490, ROR = 152.238), pleuropericarditis (PRR = 81.172, ROR = 84.970), necrotizing esophagitis (PRR = 79.842, ROR = 83.581), tooth socket hemorrhage (PRR = 78.554, ROR = 82.106), and decreased red cell distribution width (PRR = 76.738, ROR = 80.141). Lastly, the top 5 strongest signals for ponatinib were noninfective myringitis (PRR = 504.528, ROR = 882.527), oral lichenoid reaction (PRR = 336.352, ROR = 470.743), acquired ichthyosis (PRR = 271.669, ROR = 353.151), anginal equivalent (PRR = 252.264, ROR = 320.919), and intestinal transit time abnormal (PRR = 207.747, ROR = 252.150).

## Discussion

There are several methods available for mining AE data from the FAERS database. These methods include using the programming language Ruby, the statistical environment R, and web-based services [13]. However, the use of Ruby and R may prove difficult for users when it comes to mining and analyzing the FAERS data. In contrast, OpenVigil is a web-based pharmacovigilance tool that facilitates the extraction, filtration, data mining, and disproportionality analysis of AE reports from the FAERS database [9, 14]. It is worth noting that while there are other applications for mining pharmacovigilance data, they differ from OpenVigil in terms of their data cleaning techniques [15, 16]. OpenVigil, on the other hand, has undergone successful verification by the FDA and is widely employed in pharmacovigilance studies [14, 17]. Therefore, for the purposes of the present study, OpenVigil 2.1 was utilized for the retrieval and analysis of the FAERS data.

Two disproportionality analysis methods are applied to detect any potential positive signal: frequentist analysis and Bayes analysis [18]. The frequentist analysis includes ROR and PRR, while the Bayes analysis includes Bayesian confidence propagation neural network and multi-item gamma poisson shrinker. The frequentist analysis is simple, easy to calculate and understand. However, it is very sensitive to small samples and therefore prone to producing false positive signals when the number of reports is small [19]. On the other hand, the Bayes analysis is more complex since it involves distributional assumptions and optimization of the likelihood function. In this study, to reduce bias caused by using a single algorithm, two different data mining algorithms (ROR and PRR) were employed for signal detection. A signal is considered significant when both algorithms yield positive results.

Among the five BCR-ABL1 TKIs, imatinib is associated with the largest number of AE reports and the broadest signal spectrum, which is consistent with clinical practice. This is because imatinib is the first BCR‐ABL1 TKI approved by the FDA and EMA. The higher incidence rate of CML in patients aged 45–64 years old accounted for a slightly greater proportion compared to other age groups [20]. It is worth noting that AEs were more likely to occur in males, which may be related to the higher prevalence of CML in males compared to females [20]. Although the FAERS database theoretically includes global adverse event data, a majority of the data comes from the United States. Consequently, research based on the FAERS data mainly focuses on the reporting regions of AE reports in North America [21, 22]. Analysis of the SOC level reveals that certain disorders and conditions were not detected for specific TKIs. For example, bosutinib did not exhibit endocrine disorders, immune system disorders, reproductive system and breast disorders, and surgical and medical procedures. Additionally, bosutinib and ponatinib did not show any cases of pregnancy, puerperium and perinatal conditions, while dasatinib did not exhibit any cases of psychiatric disorders and social circumstances. Furthermore, there were variabilities in the case numbers, PT numbers, and signal intensity of SOCs across individual BCR‐ABL1 TKIs. Therefore, when prescribing BCR‐ABL1 TKIs for cancer patients, it is important for clinicians to consider both the adverse reaction characteristics of each drug and the specific details of the patient.

The adverse reactions most frequently reported in the labels of BCR-ABL1 TKIs were fluid retention events, gastrointestinal toxicities, hematologic toxicities, rash, pain, hepatic toxicities, and hemorrhage [23–33]. These adverse reactions are consistent with the results of the AE signals analysis, providing credibility to this study. Among these adverse reactions, fluid retention events, such as edema, pleural effusion, and pericardial effusion, were the most common for BCR‐ABL1 TKIs. Specifically, dasatinib showed a stronger association with edema (PRR = 7.168, ROR = 7.389), pleural effusion (PRR = 33.536, ROR = 36.388), and pericardial effusion (PRR = 13.327, ROR = 13.484) compared to the other four drugs. In terms of gastrointestinal toxicities, bosutinib had a stronger association with nausea and vomiting (PRR = 3.794, ROR = 4.436) and diarrhea (PRR = 10.342, ROR = 13.789), while ponatinib exhibited a stronger association with constipation (PRR = 6.526, ROR = 6.194). As for hematologic toxicities, ponatinib showed a stronger association with thrombocytopenia (PRR = 8.154, ROR = 8.678), leukopenia (PRR = 6.161, ROR = 6.317), and anemia (PRR = 10.593, ROR = 10.703). Regarding to rash, ponatinib had a stronger signal intensity (PRR = 5.016, ROR = 5.562) compared to the other four drugs. Pain was commonly reported for BCR‐ABL1 TKIs, with dasatinib showing a stronger association (PRR = 4.013, ROR = 4.045). Hepatic toxicities were also common adverse reactions, with bosutinib showing a stronger association with aminotransferase increased (PRR = 3.963, ROR = 4.005) and nilotinib showing a stronger association with blood bilirubin increased (PRR = 10.194, ROR = 10.349). Lastly, bosutinib exhibited a stronger signal intensity for hemorrhage (PRR = 12.476, ROR = 12.507) compared to the other four drugs.

New signals were observed for each BCR-ABL1 TKI, with different distribution patterns. For imatinib, the new signals were mainly distributed in investigations (33 PTs), nervous system disorders (24 PTs), and gastrointestinal disorders (23 PTs). Nilotinib showed new signals mainly in cardiac disorders (32 PTs), investigations (31 PTs), gastrointestinal disorders (25 PTs), and vascular disorders (25 PTs). Dasatinib had new signals primarily in nervous system disorders (8 PTs), infections and infestations (5 PTs), and investigations (5 PTs). Bosutinib showed new signals primarily in gastrointestinal disorders (11 PTs), respiratory, thoracic and mediastinal disorders (8 PTs), and investigations (5 PTs). Lastly, ponatinib exhibited new signals primarily in investigations (30 PTs), infections and infestations (28 PTs), gastrointestinal disorders (19 PTs), and nervous system disorders (19 PTs). Although these new signals were not included in drug labels, they had a high correlation with the respective BCR-ABL1 TKIs. It is therefore necessary to exercise caution in clinical practice.

This study comprehensively revealed the AE signals of BCR-ABL1 TKIs based on real-world data, providing strong support for monitoring adverse drug reactions (ADRs) and rational clinical use of drugs. However, there are several limitations to this study. Firstly, the FAERS database, which was used in this analysis, is a self-reporting system with reporting randomness and massive missing data. As a result, the analysis results may deviate from the actual situation [34]. Secondly, since the FAERS database only includes cases with AEs, it cannot provide the total number of patients receiving BCR-ABL1 TKIs treatment, making it impossible to estimate the incidence rate of AEs associated with each drug [35]. Thirdly, due to the self-reporting nature of the FAERS database, it is difficult to determine the causal relationship between AEs and drugs [36]. Therefore, further studies are required to validate our findings. Finally, there is a distinction between AEs and ADRs. While ADRs are caused by drugs, AEs can be caused by drugs as well as by the disease itself or other factors. Therefore, clinicians must assess if an AE is drug-related using clinical realities. Despite these limitations, the FAERS database remains a rich resource and an important tool for post-marketing surveillance.

## Conclusion

Based on the FAERS database, we mined and analyzed the AE signals of different BCR-ABL1 TKIs in this study. The study revealed that AE signals associated with BCR-ABL1 TKIs varied. Imatinib and ponatinib mainly exhibited AE signals related to general disorders and administration site conditions. On the other hand, nilotinib, dasatinib, and bosutinib showed AE signals primarily associated with investigations, respiratory, thoracic and mediastinal disorders, and gastrointestinal disorders, respectively. Furthermore, new signals were observed for each BCR-ABL1 TKI, which have implications for clinical practice and drug monitoring. Clinical practices usually use official drug labels, and as we mentioned previously, similar studies are useful as idea generating that need to be further investigated taking into account all available evidence (review of actual reports from the database, clinical data, studies etc.).

### Electronic supplementary material

Below is the link to the electronic supplementary material.

Supplementary Material 1: The top 20 most frequently reported significant AE signals and new signals for each BCR-ABL1 TKI

## Data Availability

Data are available on the FAERS database.
