# Supplementary material for: Pharmacovigilance study of BCR-ABL1 tyrosine kinase inhibitors: a safety analysis of the FDA adverse event reporting system
Source: BMC Pharmacol Toxicol. 2024 Feb 23;25:20. doi: 10.1186/s40360-024-00741-x (PMC10885429; doi:10.1186/s40360-024-00741-x)
Supplement: Supplementary file 1 — Supplementary Material 1: The top 20 most frequently reported significant AE signals and new signals for each BCR-ABL1 TKI [file 40360_2024_741_MOESM1_ESM.docx]

Supplemental Table 1. Top 20 most frequently reported AE signals of imatinib

| PTs | N | ROR (95% CI) | PRR (χ^2^) |
| --- | --- | --- | --- |
| Death | 12257 | 7.950 (7.786-8.117) | 6.116 (53598.426) |
| Malignant neoplasm progression | 1542 | 8.512 (8.084-8.962) | 8.263 (9571.280) |
| Anaemia | 1168 | 3.188 (3.007-3.380) | 3.133 (1687.246) |
| Neoplasm malignant | 1051 | 6.059 (5.695-6.445) | 5.944 (4236.565) |
| Muscle spasms | 1029 | 2.847 (2.676-3.029) | 2.806 (1191.018) |
| Drug resistance | 912 | 18.875 (17.637-20.199) | 18.524 (14107.837) |
| Drug intolerance | 809 | 4.421 (4.122-4.742) | 4.362 (2066.354) |
| Haemoglobin decreased | 791 | 4.054 (3.777-4.352) | 4.002 (1759.018) |
| Pleural effusion | 761 | 6.358 (5.913-6.836) | 6.270 (3294.498) |
| Oedema peripheral | 750 | 2.649 (2.464-2.849) | 2.623 (748.472) |
| Second primary malignancy | 668 | 43.229 (39.811-46.94) | 42.622 (23282.443) |
| Platelet count decreased | 656 | 3.099 (2.868-3.349) | 3.070 (906.885) |
| Thrombocytopenia | 641 | 3.057 (2.826-3.306) | 3.028 (862.682) |
| Oedema | 617 | 5.721 (5.280-6.200) | 5.659 (2316.708) |
| Disease progression | 602 | 2.637 (2.432-2.859) | 2.616 (596.255) |
| White blood cell count decreased | 575 | 2.606 (2.399-2.830) | 2.586 (554.887) |
| White blood cell count increased | 555 | 7.237 (6.648-7.877) | 7.162 (2862.088) |
| Fluid retention | 549 | 5.250 (4.822-5.716) | 5.200 (1825.683) |
| Pancytopenia | 501 | 4.997 (4.572-5.462) | 4.954 (1551.025) |
| Sepsis | 459 | 2.174 (1.983-2.385) | 2.163 (284.700) |

ROR, reporting odds ratio; PRR, proportional reporting ratio; CI, confidence interval; χ^2^, chi-squared.

Supplemental Table 2. Top 20 most frequently reported AE signals of nilotinib

| PTs | N | ROR (95% CI) | PRR (χ^2^) |
| --- | --- | --- | --- |
| Death | 2454 | 2.789 (2.674-2.909) | 2.581 (2475.927) |
| Rash | 1081 | 3.126 (2.940-3.324) | 3.017 (1472.838) |
| Electrocardiogram QT prolonged | 759 | 21.613 (20.075-23.269) | 20.873 (13822.393) |
| Myocardial infarction | 749 | 3.885 (3.611-4.180) | 3.783 (1534.053) |
| Pyrexia | 668 | 2.376 (2.200-2.567) | 2.333 (512.021) |
| Pruritus | 656 | 2.387 (2.208-2.580) | 2.344 (508.521) |
| Pain in extremity | 613 | 2.292 (2.115-2.484) | 2.255 (430.459) |
| Chest pain | 567 | 3.507 (3.226-3.814) | 3.440 (980.294) |
| Platelet count decreased | 566 | 6.147 (5.652-6.685) | 6.009 (2342.377) |
| Anaemia | 531 | 3.291 (3.018-3.588) | 3.233 (818.286) |
| Cerebrovascular accident | 481 | 2.746 (2.508-3.007) | 2.706 (517.564) |
| Malignant neoplasm progression | 470 | 5.503 (5.020-6.032) | 5.403 (1671.775) |
| Abdominal pain | 459 | 2.379 (2.169-2.611) | 2.350 (356.174) |
| Arteriosclerosis | 438 | 55.738 (50.469-61.557) | 54.604 (20855.299) |
| Pleural effusion | 432 | 8.407 (7.637-9.254) | 8.255 (2712.048) |
| Abdominal pain upper | 429 | 2.401 (2.182-2.643) | 2.373 (340.754) |
| Thrombocytopenia | 420 | 4.648 (4.218-5.121) | 4.575 (1164.900) |
| Myalgia | 408 | 2.651 (2.403-2.925) | 2.620 (407.941) |
| Haemoglobin decreased | 404 | 4.823 (4.369-5.325) | 4.750 (1186.668) |
| Alopecia | 392 | 2.098 (1.898-2.319) | 2.078 (219.128) |

ROR, reporting odds ratio; PRR, proportional reporting ratio; CI, confidence interval; χ^2^, chi-squared.

Supplemental Table 3. Top 20 most frequently reported AE signals of dasatinib

| PTs | N | ROR (95% CI) | PRR (χ^2^) |
| --- | --- | --- | --- |
| Pleural effusion | 1831 | 36.388 (34.641-38.223) | 33.536 (54319.547) |
| Fatigue | 1546 | 2.158 (2.049-2.273) | 2.079 (890.721) |
| Diarrhoea | 1296 | 2.287 (2.162-2.419) | 2.213 (879.959) |
| Headache | 1287 | 2.125 (2.008-2.248) | 2.061 (718.721) |
| Rash | 958 | 2.546 (2.386-2.716) | 2.480 (855.461) |
| Hospitalisation | 803 | 5.381 (5.013-5.775) | 5.226 (2730.474) |
| Pulmonary oedema | 564 | 14.033 (12.894-15.273) | 13.710 (6470.039) |
| Hepatotoxicity | 537 | 23.892 (21.890-26.076) | 23.350 (10972.609) |
| Malignant neoplasm progression | 489 | 5.357 (4.896-5.862) | 5.264 (1674.114) |
| Fluid retention | 414 | 8.141 (7.382-8.979) | 8.011 (2499.689) |
| Myalgia | 345 | 2.046 (1.840-2.277) | 2.031 (180.047) |
| Pericardial effusion | 286 | 13.484 (11.981-15.175) | 13.327 (3168.379) |
| Platelet count decreased | 278 | 2.729 (2.423-3.072) | 2.707 (297.398) |
| Bone pain | 233 | 4.028 (3.538-4.585) | 3.997 (517.795) |
| White blood cell count decreased | 229 | 2.146 (1.883-2.445) | 2.134 (136.977) |
| Thrombocytopenia | 227 | 2.268 (1.990-2.586) | 2.256 (157.423) |
| Pulmonary hypertension | 163 | 7.240 (6.199-8.455) | 7.195 (852.045) |
| Acne | 159 | 4.775 (4.083-5.586) | 4.749 (463.187) |
| Oedema | 152 | 2.932 (2.499-3.441) | 2.919 (189.243) |
| Pancytopenia | 131 | 2.727 (2.296-3.239) | 2.717 (139.984) |

ROR, reporting odds ratio; PRR, proportional reporting ratio; CI, confidence interval; χ^2^, chi-squared.

Supplemental Table 4. Top 20 most frequently reported AE signals of bosutinib

| PTs | N | ROR (95% CI) | PRR (χ^2^) |
| --- | --- | --- | --- |
| Diarrhoea | 1321 | 13.789 (12.944-14.689) | 10.342 (11373.007) |
| Nausea | 589 | 4.204 (3.857-4.582) | 3.819 (1259.955) |
| Fatigue | 375 | 2.408 (2.167-2.675) | 2.300 (283.349) |
| Vomiting | 327 | 3.946 (3.526-4.415) | 3.749 (666.963) |
| Rash | 270 | 3.366 (2.977-3.806) | 3.236 (421.389) |
| Neoplasm progression | 266 | 29.144 (25.733-33.006) | 27.616 (6709.041) |
| Malaise | 210 | 2.059 (1.793-2.365) | 2.014 (108.353) |
| Abdominal pain upper | 145 | 3.719 (3.152-4.388) | 3.639 (276.526) |
| Abdominal pain | 133 | 3.466 (2.917-4.119) | 3.399 (224.217) |
| Pleural effusion | 125 | 12.018 (10.057-14.362) | 11.737 (1211.958) |
| Weight decreased | 121 | 2.447 (2.042-2.931) | 2.411 (99.382) |
| Decreased appetite | 119 | 2.756 (2.298-3.306) | 2.714 (28.019) |
| Constipation | 108 | 2.679 (2.214-3.243) | 2.642 (109.365) |
| Abdominal discomfort | 102 | 2.801 (2.302-3.408) | 2.763 (113.708) |
| Illness | 77 | 4.956 (3.956-6.209) | 4.894 (234.790) |
| Platelet count decreased | 77 | 3.632 (2.899-4.550) | 3.590 (141.671) |
| Dehydration | 76 | 3.602 (2.871-4.519) | 3.562 (137.828) |
| Second primary malignancy | 69 | 27.459 (21.613-34.885) | 27.086 (1683.415) |
| Drug intolerance | 66 | 2.791 (2.189-3.559) | 2.767 (72.951) |
| Fluid retention | 64 | 5.711 (4.461-7.312) | 5.650 (240.109) |

ROR, reporting odds ratio; PRR, proportional reporting ratio; CI, confidence interval; χ^2^, chi-squared.

Supplemental Table 5. Top 20 most frequently reported AE signals of ponatinib

| PTs | N | ROR (95% CI) | PRR (χ^2^) |
| --- | --- | --- | --- |
| Death | 1149 | 4.007 (3.763-4.267) | 3.547 (2186.695) |
| Fatigue | 646 | 2.737 (2.525-2.968) | 2.588 (647.956) |
| Rash | 591 | 4.959 (4.558-5.394) | 4.647 (1710.332) |
| Headache | 495 | 2.590 (2.364-2.837) | 2.485 (448.729) |
| Hospitalisation | 480 | 8.607 (7.844-9.443) | 8.120 (2992.916) |
| Dry skin | 417 | 20.299 (18.376-22.423) | 19.227 (7092.160) |
| Constipation | 409 | 6.845 (6.194-7.564) | 6.526 (1913.851) |
| Pyrexia | 392 | 4.515 (4.077-4.999) | 4.331 (1009.483) |
| Arthralgia | 384 | 3.373 (3.043-3.738) | 3.251 (604.276) |
| Hypertension | 374 | 6.691 (6.029-7.427) | 6.408 (1705.594) |
| Platelet count decreased | 330 | 10.515 (9.412-11.747) | 10.096 (2684.245) |
| Blood pressure increased | 302 | 7.021 (6.256-7.881) | 6.779 (1482.393) |
| Chest pain | 276 | 6.010 (5.327-6.779) | 5.825 (1099.934) |
| Neoplasm progression | 276 | 19.342 (17.135-21.832) | 18.667 (4534.497) |
| Pain in extremity | 270 | 3.105 (2.749-3.506) | 3.029 (368.416) |
| Pneumonia | 268 | 3.091 (2.735-3.492) | 3.016 (362.502) |
| Asthenia | 251 | 2.478 (2.185-2.811) | 2.429 (212.047) |
| Back pain | 244 | 3.688 (3.246-4.191) | 3.601 (458.544) |
| Myalgia | 223 | 4.567 (3.996-5.219) | 4.461 (597.095) |
| Abdominal pain | 222 | 3.794 (3.319-4.337) | 3.712 (439.248) |

ROR, reporting odds ratio; PRR, proportional reporting ratio; CI, confidence interval; χ^2^, chi-squared.

Supplemental Table 6. The new signals for each BCR‐ABL1 TKI

| SOC | Imatinib  PTs (N) | Nilotinib  PTs (N) | Dasatinib  PTs (N) | Bosutinib  PTs (N) | Ponatinib  PTs (N) |
| --- | --- | --- | --- | --- | --- |
| Blood and lymphatic system disorders | Bone marrow necrosis (33), Bone marrow oedema (24), Bone marrow reticulin fibrosis (13), Disseminated intravascular coagulation (116), Granulocytes maturation arrest (3), Hypergammaglobulinaemia (5), Hyperviscosity syndrome (8), Platelet dysfunction (8), Splenitis (4) | Bone marrow reticulin fibrosis (5), Leukostasis syndrome (3), Lymphadenitis (6), Spleen disorder (10), Splenic infarction (5), Splenitis (36), Splenomegaly (157) | Bone marrow necrosis (7), Splenomegaly (40) | Spleen disorder (4) | Anaemia folate deficiency (3), Anaemia vitamin B12 deficiency (3), Bone marrow necrosis (3), Disseminated intravascular coagulation (27), Hypergammaglobulinaemia (3), Polycythaemia (6) |
| Cardiac disorders | Adams-Stokes syndrome (3), Bundle branch block left (19), Cardiopulmonary failure (21), Heart valve incompetence (14), Mitral valve stenosis (5), Pleuropericarditis (3) | Aortic valve calcification (4), Aortic valve incompetence (18), Aortic valve sclerosis (5), Aortic valve stenosis (11), Aortic valve thickening (3), Arteriospasm coronary (17), Bundle branch block right (16), Cardiac hypertrophy (12), Cardiac tamponade (22), Cardiomegaly (61), Cardio-respiratory arrest (97), Coronary artery dilatation (8), Coronary artery thrombosis (5), Defect conduction intraventricular (3), Dilatation atrial (7), Dilatation ventricular (8), Heart valve incompetence (9), Heart valve stenosis (7), Left atrial dilatation (12), Left atrial enlargement (16), Left ventricular dilatation (3), Left ventricular hypertrophy (48), Mitral valve calcification (4), Mitral valve disease (9), Mitral valve incompetence (75), Mitral valve stenosis (7), Pericardial haemorrhage (8), Pulmonary valve incompetence (10), Restrictive cardiomyopathy (3), Right atrial dilatation (9), Sinus node dysfunction (12), Tricuspid valve incompetence (57) | Cardiac tamponade (16) | Atrial fibrillation (41), Pleuropericarditis (4) | Anginal equivalent (3), Aortic valve incompetence (9), Aortic valve sclerosis (3), Bundle branch block (3), Cardiac valve disease (7), Diastolic dysfunction (5), Mitral valve incompetence (13), Myocardial ischaemia (15), Pericarditis (16) |
| Congenital, familial and genetic disorders | Aplasia (23), Craniosynostosis (8), Hydrocele (10), Neurofibromatosis (12), Urethral valves (6) | Chronic granulomatous disease (3), Turner's syndrome (3) | Encephalocele (3) |  | Arnold-Chiari malformation (4), Ichthyosis (13) |
| Ear and labyrinth disorders | Eustachian tube disorder (3), Otorrhoea (15) | Auricular swelling (3), Cerumen impaction (9), Deafness (75), Ear congestion (7), Ear haemorrhage (9), Otorrhoea (8) | Otorrhoea (5) | Auditory disorder (5), Ear swelling (4), Hypoacusis (58) | Deafness (28), Deafness unilateral (5), Ear discomfort (17), Ear haemorrhage (5), Ear pain (16), Eustachian tube dysfunction (5), Inner ear disorder (3), Noninfective myringitis (3) |
| Endocrine disorders | Adrenomegaly (3), Pituitary apoplexy (12), Polyglandular disorder (3) | Goitre (16), Hypogonadism (5), Toxic goitre (3) |  |  | Adrenal cyst (3), Cushingoid (9), Thyrotoxic crisis (3) |
| Eye disorders | Amaurosis (6), Blindness (193), Conjunctival oedema (8), Exophthalmos (14), Lacrimal disorder (5), Lagophthalmos (13), Ocular hyperaemia (4), Optic nerve disorder (11), Optic neuropathy (17), Retinopathy (20), Sudden visual loss (3), Vogt-Koyanagi-Harada disease (3) | Amaurosis fugax (5), Arteriosclerotic retinopathy (3), Chalazion (4), Eye colour change (5), Fuchs' syndrome (4), Lacrimal disorder (9), Retinal detachment (27) |  | Blindness (25) | Cataract (38), Corneal scar (3), Diplopia (14), Eye colour change (3), Eye haemorrhage (17), Eye inflammation (8), Eyelid skin dryness (3), Halo vision (4), Keratitis (6), Night blindness (5), Ocular hypertension (5), Ophthalmoplegia (6), Photophobia (26), Retinal drusen (4), Retinal scar (4), Ulcerative keratitis (5) |
| Gastrointestinal disorders | Abdominal adhesions (16), Abdominal wall cyst (4), Dumping syndrome (3), Gastric cyst (3), Gastrointestinal angiectasia (5), Gastrointestinal erosion (5), Intestinal congestion (6), Intra-abdominal haemorrhage (33), Lip erosion (8), Megacolon (3), Oesophageal fistula (4), Oesophageal stenosis (15), Oral mucosal discolouration (9), Oral mucosal exfoliation (8), Palatal disorder (5), Pancreatic enlargement (5), Pancreatic fistula (3), Parotid gland enlargement (7), Proctitis (18), Rectal ulcer (7), Retroperitoneal fibrosis (13), Salivary gland enlargement (7), Teeth brittle (8) | Abdominal hernia obstructive (3), Abdominal rigidity (6), Brunner's gland hyperplasia (6), Coeliac artery stenosis (5), Dental plaque (3), Dyschezia (7), Enlarged uvula (3), Gastric dilatation (11), Gastrointestinal oedema (7), Gastrointestinal tract irritation (7), Gingival bleeding (25), Glossitis (8), Ileus (21), Lumbar hernia (3), Mallory-Weiss syndrome (6), Mesenteric artery stenosis (3), Mouth haemorrhage (26), Mouth swelling (18), Odynophagia (10), Oesophageal spasm (5), Pancreatic enlargement (3), Salivary gland pain (3), Tooth discolouration (7), Toothache (41), Umbilical hernia (9) | Gastric antral vascular ectasia (4), Intestinal polyp (6), Proctitis (7) | Eructation (12), Gastric ulcer (11), Gastrointestinal necrosis (3), Gastrooesophageal reflux disease (42), Gingival pain (5), Narcotic bowel syndrome (4), Necrotising oesophagitis (8), Oesophageal stenosis (3), Oesophageal ulcer (3), Tooth discolouration (3), Tooth socket haemorrhage (4) | Dental caries (10), Eructation (15), Gingival bleeding (15), Gingival erythema (5), Gingival swelling (6), Haemorrhoid (28), Hyperchlorhydria (9), Ileus (11), Intestinal obstruction (22), Lumbar hernia (3), Oesophageal irritation (3), Oesophagitis (11), Oral blood blister (3), Oral lichenoid reaction (4), Pancreatic enlargement (7), Rectal fissure (4), Small intestinal obstruction (11), Tooth discolouration (4), Tooth erosion (3) |
| General disorders and administration site conditions | Calcinosis (11), Enanthema (4), Generalised oedema (183), Multiple organ dysfunction syndrome (135), Polyserositis (15), Serositis (8) | Facial pain (20), Generalised oedema (42), Necrosis (29),  Serositis (3) | Serositis (3) | Sluggishness (7), Ulcer (10) | Axillary pain (5), Impaired healing (16), Multiple organ dysfunction syndrome (20) |
| Hepatobiliary disorders | Biliary dilatation (7), Congestive hepatopathy (3), Hepatic calcification (4), Hepatic cyst (16), Hepatic cytolysis (26), Hepatic fibrosis (17), Portal fibrosis (3), Primary biliary cholangitis (4) | Bile duct stone (11), Cholangitis acute (3), Cholecystitis (29), Cholecystitis acute (20), Cholelithiasis (114), Hepatic pain (17), Hepatosplenomegaly (6) | Hepatic vein occlusion (4), Hepatomegaly (24), Liver tenderness (3) | Cholelithiasis (12), Gallbladder disorder (7) | Cholecystitis (19), Cholecystitis acute (14), Cholelithiasis (16), Gallbladder disorder (39), Hepatic cirrhosis (18), Hepatic necrosis (4), Hepatic steatosis (10), Hepatomegaly (27) |
| Immune system disorders | Decreased immune responsiveness (47), Hypogammaglobulinaemia (27) | Decreased immune responsiveness (56) |  |  | Cell-mediated immune deficiency (3), Hypogammaglobulinaemia (16), Immune system disorder (12), Immunodeficiency (14), Immunosuppression (11), Secondary immunodeficiency (3) |
| Infections and infestations | Bone tuberculosis (3), Emphysematous cystitis (3), Gangrene (41), Gas gangrene (4), Genital abscess (3), Liver abscess (34), Lung abscess (15), Measles (6), Muscle abscess (6), Parotitis (10), Peritonsillar abscess (7), Pharyngotonsillitis (4), Prostatic abscess (4), Pulmonary tuberculosis (37), Renal tuberculosis (3), Septic shock (185), Tuberculosis (119), Typhoid fever (36) | Acute sinusitis (6), Amoebiasis (3), Chronic sinusitis (8), Dengue fever (16), Dysentery (9), Endocarditis (9), Epididymitis (5), Erysipelas (12), Eye abscess (4), Gangrene (51), Helicobacter infection (14), Hordeolum (8), Labyrinthitis (6), Lymph node tuberculosis (3), Measles (6), Osteomyelitis acute (3), Otitis externa (4), Perineal abscess (3), Peritonsillar abscess (5), Septic embolus (3), Varicella (9) | Endocarditis (10), Endocarditis staphylococcal (3), Erysipelas (10), Periodontitis (4), Splenic abscess (3) | Arthritis infective (4), Gangrene (4), Sialoadenitis (3) | Abscess limb (4), Appendicitis perforated (3), Body tinea (3), Bronchitis (51), Cholecystitis infective (6), Cystitis (24), Cystitis viral (3), Cytomegalovirus chorioretinitis (3), Folliculitis (7), Furuncle (14), Gangrene (5), Gingivitis (10), Herpes virus infection (5), Herpes zoster (45), Hordeolum (7), Keratitis fungal (3), Labyrinthitis (4), Mastitis (3), Meningitis (10), Myelitis (3), Nasal abscess (5), Parotitis (4), Periodontitis (3), Pertussis (3), Rash pustular (12), Retinitis (3), Tonsillitis (7), Tooth abscess (12) |
| Injury, poisoning and procedural complications | Anastomotic stenosis (6), Brain herniation (16), Complications of bone marrow transplant (4), Complications of transplanted heart (4), Engraft failure (3), Gastrointestinal anastomotic leak (3), Hepatic rupture (3), Post embolisation syndrome (9), Splenic rupture (19), Transplantation complication (5) | Brain herniation (10), Burn oesophageal (4), Complications of bone marrow transplant (4), Delayed effects of radiation (4), Sternal fracture (4), Vaccination complication (4), Vascular graft occlusion (3), Vascular procedure complication (4), Wound complication (6) | Sunburn (23) | Foot fracture (8) | Concussion (6), Facial bones fracture (5), Hip fracture (19), Pelvic fracture (6), Post lumbar puncture syndrome (4), Skin laceration (6), Skull fracture (3), Splenic rupture (9), Sunburn (13) |
| Investigations | Aldolase increased (5), Angiotensin converting enzyme increased (4), Blast cell count increased (48), Blood beta-D-glucan increased (3), Blood chloride increased (9), Blood cholinesterase decreased (3), Blood follicle stimulating hormone increased (11), Blood immunoglobulin A increased (4), Blood immunoglobulin G increased (7), Blood iron decreased (41), Blood mercury abnormal (3), Blood pH increased (5), Blood sodium increased (15), Blood zinc decreased (3), Carnitine decreased (3), CSF pressure increased (4), CSF test abnormal (6), Full blood count increased (49), Gamma-glutamyltransferase increased (100), Haptoglobin decreased (5), Immunoglobulins decreased (6), Leucine aminopeptidase increased (3), Mean cell volume abnormal (4), Megakaryocytes abnormal (3), Muscle enzyme increased (7), Platelet count increased (215), PO2 decreased (7), Pulmonary arterial pressure abnormal (4), Serum ferritin decreased (8), Specific gravity urine increased (3), Transferrin saturation decreased (3), Urine calcium increased (4), Vitamin B12 decreased (12) | Amino acid level increased (3), Anti-thyroid antibody positive (4), Blood immunoglobulin G increased (4), Blood iron decreased (29), Brain natriuretic peptide increased (12), Cardiac stress test abnormal (4), C-reactive protein decreased (3), C-reactive protein increased (57), Electrocardiogram repolarisation abnormality (11), Electrocardiogram ST segment depression (27), Electrocardiogram T wave amplitude decreased (5), Electrocardiogram T wave inversion (15), Epstein-Barr virus test positive (3), Globulins increased (3), Haematocrit decreased (51), Haemoglobin increased (13), Helicobacter test positive (6), High density lipoprotein increased (4), Intracardiac pressure increased (4), Manganese increased (4), Mean platelet volume increased (4), Oxygen consumption decreased (7), Pancreatic enzymes increased (17), Prothrombin time shortened (5), QRS axis abnormal (11), Red blood cell count increased (14), Total lung capacity decreased (6), Transferrin saturation decreased (8), Tryptase increased (4), Vitamin B12 decreased (7), White blood cells urine positive (4) | Blood immunoglobulin G decreased (5), Blood iron decreased (23), Helicobacter test positive (3), Prothrombin time abnormal (3), Right ventricular systolic pressure increased (13) | Blood iron decreased (6), Body surface area increased (4), Platelet count increased (28), Red cell distribution width decreased (5), Urine output decreased (8) | Activated partial thromboplastin time shortened (4), Blood chloride increased (12), Blood immunoglobulin G decreased (3), Blood insulin increased (3), Blood iron decreased (9), Blood iron increased (3), Blood lactate dehydrogenase abnormal (10), Blood lactate dehydrogenase increased (21), Blood magnesium decreased (7), Blood urea abnormal (8), Blood urea increased (19), Carbon dioxide decreased (8), Cardiac murmur (10), C-reactive protein increased (23), Electrocardiogram ST segment depression (8), Electrocardiogram T wave inversion (3), Fibrin D dimer increased (4), Gastric pH decreased (4), Heart sounds abnormal (4), High density lipoprotein decreased (3), Intestinal transit time abnormal (3), Mean cell haemoglobin increased (3), Mean cell volume increased (6), Protein total decreased (6), Red cell distribution width abnormal (4), Red cell distribution width decreased (3), Red cell distribution width increased (19), Serum ferritin increased (7), Urine output decreased (7), Vitamin D decreased (7) |
| Metabolism and nutrition disorders | Cachexia (37), Iron deficiency (16) | Cachexia (10), Diabetic complication (10), Iron deficiency (10), Vitamin B complex deficiency (3), Vitamin B12 deficiency (16), Vitamin D deficiency (25) | Hypervolaemia (6), Iron deficiency (10), Vitamin B complex deficiency (4) | Gout (8), Hypercholesterolaemia (4), Iron deficiency (3) | Diabetic complication (6), Gout (17), Haemochromatosis (4), Hypomagnesaemia (11), Iron deficiency (8), Periarthritis calcarea (3), Vitamin B12 deficiency (4), Vitamin D deficiency (10) |
| Musculoskeletal and connective tissue disorders | Bone formation decreased (8), Calcification of muscle (5), Chondrocalcinosis (5), Eosinophilic fasciitis (4), Fasciitis (12), Gouty arthritis (6), Morphoea (12), Muscle swelling (6), Myositis (51), Necrotising myositis (6), Scleroderma (29), Scleroderma-like reaction (3), Sjogren's syndrome (4), Trigger finger (15) | Cervical spinal stenosis (5), Dupuytren's contracture (6), Eosinophilic fasciitis (4), Facet joint syndrome (6), Intervertebral disc degeneration (21), Morphoea (6), Rheumatic disorder (9), Scleroderma (11), Trigger finger (10) |  | Bone pain (57), Intervertebral disc disorder (4), Intervertebral disc protrusion (10), Lumbar spinal stenosis (3), Spinal stenosis (3) | Articular calcification (3), Bone lesion (4), Intervertebral disc protrusion (13), Joint stiffness (16), Muscle swelling (3), Myositis (8), Osteochondrosis (3) |
| Neoplasms benign, malignant and unspecified (incl cysts and polyps) | Hypergammaglobulinaemia benign monoclonal (8), Marrow hyperplasia (27), Monoclonal gammopathy (5), Myelofibrosis (68), Pyogenic granuloma (6), Second primary malignancy (668) | Melanocytic naevus (14) |  | Second primary malignancy (69) | Melanocytic naevus (6), Neuroma (6), Skin papilloma (10) |
| Nervous system disorders | Amyotrophic lateral sclerosis (15), Asterixis (6), Axonal neuropathy (10), Basilar artery stenosis (4), Burning feet syndrome (3), Cerebral vasoconstriction (6), Coma hepatic (19), Decreased vibratory sense (5), Dementia Alzheimer's type (44), Dropped head syndrome (8), Gait apraxia (3), IIIrd nerve paralysis (9), Ischaemic cerebral infarction (15), Meningeal disorder (4), Myelitis transverse (8), Myelopathy (5), Pyramidal tract syndrome (4), Ruptured cerebral aneurysm (5), Sensorimotor disorder (6), Spinal cord haematoma (3), Tonic clonic movements (7), Trigeminal palsy (6), Vibratory sense increased (3), Vocal cord paralysis (11) | Apallic syndrome (3), Arachnoid cyst (4), Burning feet syndrome (9), Cauda equina syndrome (5), Central nervous system vasculitis (3), Cerebral artery thrombosis (3), Cerebral thrombosis (7), Cervical radiculopathy (5), Cranial nerve disorder (5), Intercostal neuralgia (3), Locked-in syndrome (3), Lumbar radiculopathy (5), Sciatica (29), Sensory loss (18), Spinal claudication (3), Spinal cord herniation (5), Spinal cord infarction (3), Thalamic infarction (3), Tongue paralysis (4), Tunnel vision (10), VIth nerve paralysis (7) | Burning feet syndrome (8), Coma hepatic (4), Demyelinating polyneuropathy (7), Guillain-Barre syndrome (13), Hydrocephalus (17), Hypertensive encephalopathy (4), Intracranial pressure increased (13), Meningeal disorder (3) | Cerebrovascular disorder (3), Parosmia (6) | Bell's palsy (5), Carotid arteriosclerosis (7), Carotid artery occlusion (6), Chronic inflammatory demyelinating polyradiculoneuropathy (8), Demyelination (7), Facial paralysis (29), IIIrd nerve paralysis (9), Intracranial aneurysm (5), Memory impairment (104), Monoplegia (5), Moyamoya disease (4), Myelitis transverse (6), Optic neuritis (7), Paraplegia (6), Parkinsonian rest tremor (3), Restless legs syndrome (12), Spinal cord disorder (3), Vertebral artery stenosis (6) |
| Pregnancy, puerperium and perinatal conditions | Placental disorder (11) | Premature rupture of membranes (7) |  |  |  |
| Renal and urinary disorders | Bence Jones proteinuria (5), Focal segmental glomerulosclerosis (10), Glomerular vascular disorder (3), Glomerulonephritis (13), Intercapillary glomerulosclerosis (3), Nephritis allergic (3), Nephrotic syndrome (31), Pelvi-ureteric obstruction (3), Ureteric obstruction (11), Ureterolithiasis (11), Urinary tract obstruction (4) | Calculus urinary (5), Kidney enlargement (5), Microalbuminuria (10), Nephrotic syndrome (14), Renal artery stenosis (21), Renal pain (20) | Crush syndrome (3), Nephritic syndrome (3) |  | Bladder dilatation (3), Bladder outlet obstruction (3), Bladder pain (6), Cystitis haemorrhagic (4), Hydronephrosis (7), Micturition disorder (4), Micturition urgency (12), Nephrocalcinosis (3), Nephrolithiasis (44), Nephrotic syndrome (8), Nocturia (13), Renal cyst (6), Renal pain (9), Urinary bladder haemorrhage (5), Urinary tract obstruction (4), Urine flow decreased (5) |
| Reproductive system and breast disorders | Azoospermia (8), Hydrometra (7), Nipple swelling (3), Ovarian atrophy (4), Ovarian enlargement (4) | Benign prostatic hyperplasia (20), Endometrial hyperplasia (5), Nipple pain (6), Oedema genital (7), Orchitis noninfective (3), Ovulation pain (4), Perineal cyst (3), Perineal induration (3), Perineal pain (3), Prostatomegaly (8), Scrotal oedema (5), Scrotal pain (6), Scrotal swelling (4), Testicular pain (11), Testicular swelling (14), Uterine haemorrhage (11), Vaginal ulceration (6) | Endometrial hyperplasia (4), Nipple disorder (3), Nipple pain (9) |  | Benign prostatic hyperplasia (12), Menopausal symptoms (4), Ovarian cyst (7), Pelvic pain (9), Penile burning sensation (4), Prostatic disorder (13), Prostatitis (5), Prostatomegaly (4), Testicular pain (6), Uterine disorder (4), Vulvovaginal pain (6) |
| Respiratory, thoracic and mediastinal disorders | Acute respiratory distress syndrome (78), Alveolar proteinosis (18), Alveolitis (14), Chylothorax (10), Diaphragmatic disorder (9), Diffuse alveolar damage (6), Hydrothorax (8), Laryngeal haemorrhage (4), Laryngeal oedema (33), Laryngeal stenosis (5), Lung cyst (5), Obliterative bronchiolitis (25), Pleural fibrosis (12), Pulmonary haemosiderosis (3), Pulmonary vein stenosis (3), Tonsillar ulcer (4) | Asphyxia (22), Atelectasis (31), Bronchial hyperreactivity (5), Chronic obstructive pulmonary disease (114), Chronic respiratory failure (6), Chylothorax (4), Emphysema (25), Lung infiltration (31), Nasal oedema (17), Pleural fibrosis (7), Pulmonary artery dilatation (5), Pulmonary cavitation (9), Pulmonary congestion (32), Sleep apnoea syndrome (37), Vocal cord polyp (4) | Alveolar proteinosis (12), Pleural fibrosis (6), Pulmonary haemorrhage (19), Respiratory tract congestion (35) | Atelectasis (4), Chronic obstructive pulmonary disease (21), Chylothorax (5), Haemoptysis (11), Lung opacity (3), Paranasal sinus hypersecretion (6), Pleurisy (4), Snoring (5) | Atelectasis (13), Bronchial hyperreactivity (5), Dry throat (7), Hypoxia (20), Idiopathic pneumonia syndrome (4), Lung infiltration (8), Nasal oedema (5), Obliterative bronchiolitis (7), Pharyngeal erythema (6), Pleuritic pain (5), Pulmonary granuloma (6), Pulmonary oedema (34), Rales (9), Snoring (5), Vocal cord dysfunction (4) |
| Skin and subcutaneous tissue disorders | Acanthosis (6), Chloasma (6), Excessive granulation tissue (6), Haemorrhage subcutaneous (20), Hypertrichosis (10), Lichenification (4), Miliaria (5), Oculomucocutaneous syndrome (4), Palmoplantar keratoderma (3), Parakeratosis (12), Parapsoriasis (3), Pityriasis rosea (5), Pyoderma gangrenosum (23), Skin atrophy (23), Skin fissures (8), Skin fragility (29), Subcutaneous emphysema (10), Vitiligo (14) | Anhidrosis (3), Dandruff (7), Diabetic foot (12), Granuloma skin (11), Lichenoid keratosis (6), Nail discolouration (10), Nail disorder (13), Pseudofolliculitis (3), Skin fissures (21), Skin fragility (4), Skin necrosis (10), Skin wrinkling (7), Solar lentigo (5), Transient acantholytic dermatosis (3), Xanthelasma (6), Xeroderma (4) | Epidermolysis (3), Skin depigmentation (7) | Miliaria (3) | Acne (34), Decubitus ulcer (6), Dermatitis acneiform (5), Eczema asteatotic (3), Ichthyosis acquired (6), Lichenification (4), Lichenoid keratosis (3), Miliaria (4), Palisaded neutrophilic granulomatous dermatitis (3), Petechiae (10), Pityriasis rubra pilaris (4), Rash follicular (7), Rash maculo-papular (32), Rash vesicular (13), Skin atrophy (5), Skin burning sensation (14), Skin hypertrophy (6), Skin wrinkling (42) |
| Surgical and medical procedures | Pleurodesis (6) | Cardioversion (5), Intestinal adhesion lysis (3) |  |  |  |
| Vascular disorders | Aneurysm ruptured (6), Aortic stenosis (17), Capillary fragility (5), Capillary leak syndrome (14), Erythrocyanosis (4), Inferior vena caval occlusion (3), Intermittent claudication (11), Vascular compression (4), Vascular fragility (4), Vascular stenosis (4) | Aortic arteriosclerosis (5), Aortic stenosis (25), Aortitis (3), Arteritis (7), Bloody discharge (6), Diabetic vascular disorder (4), Dry gangrene (10), Erythromelalgia (3), Giant cell arteritis (9), Iliac artery stenosis (5), Pallor (58), Peripheral artery thrombosis (9), Peripheral venous disease (11), Phlebitis (10), Polyarteritis nodosa (4), Raynaud's phenomenon (13), Spider vein (3), Varicose vein (16), Vascular calcification (8), Vascular compression (3), Vascular pain (5), Vascular rupture (7), Vasculitis (28), Venous occlusion (9) | Capillary leak syndrome (6), Spider vein (3) | Aortic aneurysm rupture (3), Capillary leak syndrome (3), Peripheral arterial occlusive disease (8) | Aortic arteriosclerosis (4), Arteriosclerosis (15), Cyanosis (8), Pallor (32), Raynaud's phenomenon (10), Shock haemorrhagic (6), Subclavian artery stenosis (4) |
| Psychiatric disorders |  | Compulsions (7), Feeling of despair (13), Laziness (7) |  | Thinking abnormal (9) | Adjustment disorder with depressed mood (5), Panic reaction (6) |
| Social circumstances |  | Sitting disability (3) |  |  |  |
